# Genetic assessment of hyperuricemia and gout in Asian, Native Hawaiian, and Pacific Islander subgroups of pregnant women: biospecimens repository cross-sectional study

**DOI:** 10.1186/s41927-021-00239-7

**Published:** 2022-01-06

**Authors:** Ali Alghubayshi, Alison Edelman, Khalifa Alrajeh, Youssef Roman

**Affiliations:** 1 Department of Pharmacotherapy and Outcomes Science, School of Pharmacy, Virginia Commonwealth University, Richmond, VA 23298-0533 USA; 2 Department of Obstetrics and Gynecology, School of Medicine, Oregon Health Science University, Portland, OR 97239 USA

**Keywords:** Gout, Hyperuricemia, Health disparities, Asian subgroups, Native Hawaiian, Pacific Islanders, Single nucleotide polymorphisms, Pregnancy, Race, Ethnicity

## Abstract

**Background:**

Gout, an inflammatory condition, is characterized by the precipitation of monosodium urate crystals (MSU) in or around distal joints. The latter is caused by chronic hyperuricemia (HU)—high urate levels in the blood. Genetic variations in urate transporters play a significant role in determining urate levels within the human body, rendering some racial and ethnic groups more or less susceptible to developing either HU or gout. This study aims to estimate the frequencies of HU and gout risk alleles in Asian, Native Hawaiian, and Pacific Islander subgroups, using biorepository DNA samples.

**Methods:**

The biospecimens repository at the University of Hawai’i provided DNA samples of consented post-partum women of Japanese, Filipino, Korean, Native Hawaiian, Samoan, and Marshallese descent. The DNA was previously extracted from maternal blood and genotyped at the Genomics and Bioinformatics Shared Resource, Cancer Center (Honolulu, HI). Nine urate genes: *ABCG2, SLC2A9, SLC16A9, GCKR, SLC22A11, SLC22A12, LRR16A, PDZK1, and SLC17A1*, were selected due to their significant association with HU and gout risk. Hardy–Weinberg Equilibrium (HWE) for genotype frequencies was assessed, using the Chi-Square test with *p* < 0.006 for statistical significance. Allele frequencies in our study were then compared to EUR from the 1000 Genomes Project Database Phase III, using Chi-square or Fisher's exact test, when appropriate. Bonferroni correction for multiple comparisons was used, with *p* < 0.006 for statistical significance.

**Results:**

Our study involved 1059 post-partum women 18-year-old or older who self-reported their respective race and ethnicity, including Asian, Native Hawaiian, and Pacific Islander ancestry. The Asian subgroups included Japanese, Filipino, and Korean. The Pacific Islander subgroups included Marshallese and Samoan. None of the study participants had a history of gout. We excluded the *PDZK1* gene from the final analysis due to its deviation from HWE (*p* < 0.006) across all the population subgroups, with eight loci remaining for cross-subgroup comparisons. Compared to EUR, the genetic polymorphism frequencies were significantly different-8/8 in Japanese, 6/8 in Korean, 6/8 in Filipino, 8/8 in Samoan, 6/8 in Native Hawaiian, and 6/8 in Marshallese. HU and gout risk alleles indices were 8, 6, 5, 5, 4, and 4 in Japanese, Filipino, Korean, Samoan, Marshallese, and Native Hawaiian, respectively. The percentage of cumulative risk alleles was 100% in both Japanese and Filipino, followed by 83.5% in Korean.

**Conclusions:**

Compared to EUR, Asian subgroups, particularly Japanese, Filipino, and Korean, had the highest percentage of the cumulative uric acid risk alleles. These results could partly explain the increased risk of developing gout among some Asian ancestral subgroups compared to EUR.

## Background

Gout is one of the most common inflammatory arthritic conditions characterized by the precipitation of monosodium urate crystals (MSU) in or around distal joints [1]. Chronically elevated serum urate (SU), a condition known as hyperuricemia (HU), is the culprit of developing gout. Acute gout flare affects monoarticular joints (e.g., knee, ankle, and metatarsophalangeal), causing severe inflammation with excruciating pain, swelling, erythema, and reduced mobility [2] The prevalence of gout in developed countries is higher than the developing ones. In the United States (US), gout prevalence is up to 3.9% affecting about 9.2 million people. Gout and hyperuricemia prevalence varies by sex and age groups. Also, specific racial and ethnic subgroups tend to have distinct HU and gout prevalence, ushering the notion of population-specific risk and suggesting distinct HU and gout allele frequencies across different racial and ethnic groups.

Many factors play different roles in regulating SU levels and might lead to HU and gout [3]. Genetic polymorphisms in urate transporters, mainly single nucleotide polymorphisms (SNPs), have been implicated in developing HU or gout. Numerous studies have ascertained the role of the genetic variation of urate transporters, and it estimated the heritability of urate by up to 73% [4]. One of the largest genome-wide association studies (GWAS) metanalysis, involving more than 110,000 participants from different racial backgrounds, discovered 28 loci associated with SU levels [5]. These loci are predominately in genes encoding urate transporters, including *SLC2A9, ABCG2, SLC22A11, SLC22A12, SLC17A1,* and the scaffolding protein-encoding gene *PDZK1* [4].

Indeed, the prevalence of HU and gout varies among people and countries. Along with differences in the genetic background, several demographic and environmental characteristics such as diet and lifestyle, smoking, alcohol consumption, or beverages containing high amounts of fructose corn syrup may increase HU and gout prevalence [6]. Studies published since 2015 showed a substantial increase in gout incidence over recent decades in the US, Canada, Denmark, Sweden, and South Korea, confirming greater incidence in men relative to women and increased incidence in later life decades. Also, recent studies in North America and Scandinavia found a 1.5–twofold increase in gout incidence over the past two to three decades [7–12]. Gout incidence in South Korea increased by 25% between 2009 and 2015 [13]. A recent study reported that the Maori and Pacific Islanders populations in New Zealand have a gout prevalence of 7.63% [14]. These trends indicate that gout incidence increased in many countries over recent decades and that the aging population in these countries may drive this increased gout incidence. Gout prevalence varies globally, with one of the highest prevalence in Oceanic countries, particularly in indigenous and South Pacific Island populations. Along with the rising gout prevalence earlier reported in Europe and the US, there is increasing evidence of growing prevalence in Australia (self-reported), Canada, China, and South Korea [15]. According to the US Census Bureau, Chinese and Filipino groups are considered two of the largest Asian subgroups. Similarly, Native Hawaiian and Samoan are the largest Pacific Islander subgroups [16]. Amongst all the ethnic subgroups in the US, populations with Asian ancestry are approximately three times more likely to develop gout than Europeans (EUR) [17].

There is an association between genetic polymorphisms in urate disposition and the incidence of gout amongst different ethnic groups. Therefore, the purpose of this study is to estimate the frequencies of selected SNPs in specific urate genes across diverse populations (Filipino, Japanese, Korean, Samoan, Marshallese, and Native Hawaiian) compared with the EUR population. With the growing need for racial diversity in genomic research, this study will further our understanding of the genetics of HU and gout in underrepresented population subgroups. We hypothesized that the risk allele frequencies of HU and gout are significantly higher in the Asian, Native Hawaiian, and Pacific Islander subgroups compared with the EUR population.

## Methods

### Study participants and urate genes

Participants included in this study were pregnant women aged 18-year-old or older. All participants self-reported 100% of their respective race/ethnicity, indicated by both biological parents and four grandparents being of the same race/ethnicity. We excluded participants with a history of cancer or organ transplant. The uric acid gene/SNPs pairs included: *SLC17A1* (rs1183201), *PDZK1* (rs12129861), *SLC22A11* (rs17300741), *ABCG2* (rs2231142), *SLC16A9* (rs2242206), *SLC22A12* (rs505802), *SLC2A9* (rs734553), *LRRC16A* (rs742132), *GCKR* (rs780094).

### Sample procurement and genotyping

DNA samples with limited medical and demographics information of study participants were provided by the University of Hawai’i biospecimens repository. Historically, these samples were collected after obtaining written consent. The placenta and umbilical cord of the participants were collected as part of the routine care. DNA extraction was conducted on maternal blood samples, and genotyping was carried out at the Genomics and Bioinformatics Shared Resource, Cancer Center (Honolulu, HI). A customized TaqMan genotyping assay panel was run on the Quant Studio 12K Flex Real-Time PCR system (Applied Biosystems). All study details were previously published [18]. All study materials were reviewed and exempted by the University of Hawai’i Human Studies Program (Protocol Number: 2018-00225).

### Statistical analysis

Continuous variables were summarized as means (± standard deviation); categorical variables were summarized as counts and percentages (%). Allele frequencies of our population subgroups were compared with EUR, using Chi-square or Fisher’s exact test, when appropriate. Bonferroni correction was used for multiple comparisons with *p* < 0.006 for statistical significance. Deviation from Hardy–Weinberg Equilibrium in our selected genetic polymorphisms was assessed using Chi-square test with *p* < 0.006 for statistical significance. Ensemble genome browser was used to estimate the allele and genotype frequencies of the EUR population (Referent group). In our analysis, we defined the urate risk allele as the allele associated with baseline or higher risks of developing HU or gout. The data analysis was performed using SPSS Statistics for Windows, version 23 (IBM SPSS, IBM Corp., Armonk, N.Y., USA).

## Results

### Study participants characteristics and demographics

In this study, 1059 participants were included. Demographic characteristics of all participants are shown in Table 1. The participant’s age ranged from 18 to 47 years with a means of 29 years. The gestational age ranged from 24 to 41 weeks with a means of 38 weeks, of which 82.2% (n = 871) were full term and 17.3% (n = 182) were pre-term. Using the pregravida weight, the body mass index (BMI) ranged from 24.5 to 30.1 kg/m^2^, with a means of 26.3 kg/m^2^, of which 43.4% (n = 400) were classified as having normal weight, 26.9% (n = 248) were classified as obese, 22.9% (n = 211) were classified as overweight, and 6.8% (n = 63) were classified as underweight. Our study consisted of 21.5%. (n = 229) Filipinos, 19.8% (n = 210) Japanese, 18.9% (n = 200) Samoans, 15.1% (n = 160) Marshallese, 14.7% (n = 156) Native Hawaiian, and 9.8% (n = 104) were Koreans. No subjects reported a history of gout.

**Table 1:** Demographic characteristics across ancestral subgroups

Characteristics	Total population (n = 1059)	Filipino (n = 229)	Japanese (n = 210)	Samoan (n = 200)	Marshallese (n = 160)	Native Hawaiian (n = 156)	Korean (n = 104)
Mother’s age (years)	28.8 ± 6.3	29.8 ± 6.1	33.4 ± 5.2	26.3 ± 5.7	25.1 ± 4.6	26.2 ± 5.5	31.3 ± 5.2
Gestational age (weeks)	38.0 ± 2.2	37.8 ± 2.2	37.6 ± 2.6	38.4 ± 2.0	38.0 ± 2.0	38.2 ± 2.0	38.3 ± 2.4
Gestational age category							
Preterm (< 37 weeks)	182 (17.3%)	44 (19.4%)	50 (23.8%)	27 (13.5%)	29 (18.4%)	21 (13.5%)	11 (10.8%)
Full term (≥ 37 weeks)	871 (82.2%)	183 (80.6%)	160 (76.2%)	173 (86.5%)	129 (81.6%)	135 (86.5%)	91 (89.2%)
Body mass index (kg/m^2^)	26.3 ± 6.9	25.0 ± 6.0	24.4 ± 5.6	30.1 ± 7.5	25.1 ± 6.2	28.1 ± 7.3	24.5 ± 7.2
Body mass index categories							
Underweight (< 18.5 kg/m^2^)	63 (6.8%)	18 (9.0%)	20 (10.4%)	5 (2.9%)	10 (7.8%)	–	10 (11.5%)
Normal weight (18.5–24.9 kg/m^2^)	400 (43.4%)	94 (46.8%)	93 (48.4%)	44 (25.3%)	68 (53.1%)	58 (41.4%)	43 (49.4%)
Overweight (25–29.9 kg/m^2^)	211 (22.9%)	56 (27.9%)	46 (24.0%)	38 (21.8%)	18 (14.1%)	30 (21.4%)	23 (26.4%)
Obese (≥ 30 kg/m^2^)	248 (26.9%)	33 (16.4%)	33 (17.2%)	87 (50.0%)	32 (25.0%)	52 (37.1%)	11 (12.6%)
Pre- gravida weight (Ibs)	151.2 ± 46.5	132.7 ± 28.4	127.2 ± 26.0	203.7 ± 44.7	142.5 ± 41.9	166.9 ± 47.9	133.6 ± 34.5

Data are expressed as count (%) or means + / − standard deviation

### Hyperuricemia and gout risk alleles frequencies

Table 2 describes the role of select SNPs within specific uric acid genes. Risk alleles and genotype frequencies of all nine uric acid genes/SNP pairs in all ethnic subgroups are summarized in Tables 3 and 4. Briefly, the prevalence of the T allele of rs2231142 (G > **T) in** *ABCG2* was 9.4% in EUR, 45.8% in Filipinos, 27.8% in Koreans, and 25.6% in Japanese. Furthermore, in the NHPI subgroups, the frequencies of the T allele were 31.1%, 17.6%, and 12.7% in Samoan, Marshallese, and Native Hawaiian, respectively. The T allele of the rs734553 (G > **T**) in *SLC2A9* was higher in Asian and Pacific Islander population subgroups than in EUR. Specifically, the frequency of the T allele of the rs734553 (G > **T**) was 99.5% in Japanese, 98.8% in Filipinos, and 98.3% in Koreans compared to 75.5% in EUR. Additionally, the frequency of rs734553 (G > **T**) was 100% in Marshallese, 98.3% in Samoan, and 90.9% in Native Hawaiians compared to 75.5% in EUR.

**Table 2:** Gene (SNP) and function summary

Gene (Protein)	Protein Function	SNP (Class)	SNP Effect	References
*ABCG2* (ABCG2)	One of the most important UA regulatory transporter. The ATP-binding cassette transporter is responsible for urate excretion and expressed kidneys, GI tract, and liver	rs2231142 (G > **T**) (Missense variant)	Reduction in ABCG2 transporter activity by 50%; urate under-excretion and hyperuricemia are caused by Glu 141 Lys amino acid substitution	[34]
*SLC2A9* (GLUT9)	High-capacity urate, fructose, and glucose transporter located on both sides of the kidney’s apical and basolateral membrane. This protein is expressed in liver, kidney, and chondrocytes tissues. Also strongly associated with increase serum UA	rs734553 (G > **T**) (Intronic variant)	Increases risk for gout through altering urate transporter affinity	[52]
*SLC16A9* (MCT9)	Monocarboxylic acid transporter protein located in the apical side of kidneys and is responsible for urate excretion	rs2242206 (G > **T**) (Missense variant)	Reported to substantially increase the risk of renal overload gout with an odds ratio of 1.28 (*p* = 0.012)	[42]
*SLC17A1* (NPT1)	Uric acid transport protein located on the apical membrane of the renal proximal convoluted tubule and contributes to urate efflux	rs1183201(**T** > A) (Intronic variant)	It is known to be associated with decreased urate levels and the A allele seems to be the protective allele in the EUR population (Beta effect = − 0.062)	[34]
*SLC22A11* (OAT4)	Organic anion transporter 4 (OAT4) is a UA transporter and is responsible for urate reabsorption through the kidney	rs17300741(G > **A**) (Intronic variant)	It is linked to renal under-excretion of SU in EUR descent (Beta effect = 0.062)	[34, 40]
*SLC22A12* (URAT1)	Urate 1 transporter (URAT1) is an important UA transporter located on the apical side of the proximal tubule and is responsible for the majority of UA reabsorption	rs505802 (**C** > T) (Intergenic variant)	It is associated with decreased SU levels in the EUR population. (Beta effect = − 0.056)	[34]
*GCKR* (GCKR)	Glucokinase is a regulator protein with a role in endocrine and metabolic phenotypes that may be associated with urate concentrations	rs780094 (C > **T**) (Intronic variant)	It is associated with glucose metabolism, lipid levels, gout risk, and SU levels (Beta effect = 0.052)	[34, 43, 44]
*PDZK1* (PDZ)	It is a scaffolding protein located on the apical side of the proximal tubule in the kidney, which has a role in maintaining the balance of urate levels through the formation of urate transportome	rs12129861(**C** > T) (Intergenic variant)	It is associated with lower serum urate levels among people of EUR ancestry (Beta effect = − 0.06)	[34, 53]
*LRRC16A* (CARMIL1)	A protein expressed on the apical side of proximal tubules in the kidneys, which is known as capping protein ARP2/3 and myosin-I linker (CARMIL). This protein has a role in urate transportome formation, which mediates UA reabsorption	rs742132 (G > **A)** (Intronic variant)	The risk allele A is associated with increased SU in individuals of EUR descent (Beta effect = 0.054)	[34, 46]

**Table 3:** Uric acid risk allele frequencies comparisons Asian and Native Hawaiian and Pacific Islanders Subgroups

Gene (SNP)	Variant Type	Allele	EUR† % (n)	Filipino % (n)	Korean % (n)	Japanese % (n)	Native Hawaiian % (n)	Marshallese % (n)	Samoan % (n)	Gout/ Urate Effect (↑↓)
*ABCG2* (rs2231142 G > **T**)	Missense	G **T**	90.6 (911) **9.4 (95)**	54.2 (194)* **45.8 (164)**	72.2 (133)* **27.8 (51)**	74.4 (278) * **25.6 (96)**	87.3 (253) **12.7(37)**	82.4 (201)* **17.6 (43)**	68.9 (251)* **31.1 (113)**	**↑**
*SLC2A9* (rs734553 G > **T**)	Intronic	G **T**	24.5 (246) **75.5 (760)**	1.2 (4)* **98.8 (348)**	1.7 (3)* **98.3 (183)**	0.5 (2)* **99.5 (368)**	9.1 (26)* **90.9 (260)**	0.0 (0)* **100.0 (242)**	1.7 (6)* **98.3 (358)**	**↑**
*SLC17A1* (rs1183201**T** > A)	Intronic	A **T**	46.1 (464) **53.9 (542)**	20.9 (73) * **79.1 (277)**	14.6 (26)* **85.4 (152)**	17.1 (63)* **82.9 (305)**	34.0 (98)* **66.0 (190)**	57.2 (135)* **42.8 (101)**	28.4(103)* **71.6 (259)**	**↓**
*SLC16A9* (s2242206 G > **T**)	Missense	G **T**	73.4 (738) **26.6 (268)**	55.0 (197)* **45.0 (161)**	40.8 (75) * **59.2 (109)**	44.5 (163)* **55.5 (203)**	54.9(158)* **45.1 (130)**	55.5 (132)* **44.5 (238)**	60.7(221)* **39.3 (143)**	**↓**
*GCKR* (rs780094 C > **T**)	Intronic	C **T**	58.9 (593) **41.1 (413)**	55.1 (197) **44.9 (161)**	58.2 (107) **41.8 (77)**	42.0 (156) * **58.0 (216)**	65.9 (190) **34.1 (98)**	64.5 (156) **35.5 (86)**	69.4(254)* **30.6 (112)**	**↑**
*SLC22A11 * (rs17300741G** > A**)	Intronic	**A** G	**46.2 (465)** 53.8 (541)	85.3 (297)* 14.7 (51)	89.6 (163)* 10.4 (19)	84.7(305) * 15.3 (55)	72.3 (201) * **27.7 (77)**	70.0 (167) * 30.0 (73)	78.5 (281)* 21.5 (77)	**↑**
*SLC22A12* (rs505802 **C** > T**)**	Intergenic	T **C**	70.7 (711) **29.3 (295)**	21.6 (79)* **78.4 (287)**	20.4 (38)* **79.6 (148)**	18.3 (68)* **81.7 (304)**	37.6 (109)* **62.4 (181)**	5.0 (12) * **95.0 (230)**	31.5(116)* **68.5 (252)**	↓
*LRRC16A* (rs742132 G > **A)**	Intronic	**A** G	**69.0 (694)** 31.0 (312)	**69.7 (251)** 30.3 (109)	**78.0 (142)** 22.0 (40)	78.2 (291)* 21.8 (81)	58.6 (171)* 41.4 (121)	**70.0 (168)** 30.0 (72)	51.7 (188)* 48.3 (176)	**↑**
PDZK1** (rs12129861 **C** > T)	Intergenic	**C** T	**54.1 (544)** 45.9 (462)	44.7 (151)* 55.3 (187)	**56.8 (92)** 43.2 (70)	**48.9 (178)** 51.1 (186)	39.3 (106)* 60.7 (164)	39.5 (90)* 60.5 (138)	46.5 (159)* 53.5 (183)	↓

The bolded letter refers to the risk allele linked to hyperuricemia or gout

^*^Indicates statistical significance *p* < 0.006 between the subgroups and the comparator group (EUR)

^†^Data from the 1000 Genomes Project Phase III

^**^Allele deviated from HWE in all study subgroups

**Table 4:** Uric acid Genotype frequencies comparisons Asian, Native Hawaiian, and Pacific Islanders Subgroups

Gene (SNP)	Genotype	EUR† % (n)	Filipino % (n)	Korean % (n)	Japanese % (n)	Native Hawaiian % (n)	Marshallese % (n)	Samoan % (n)
*ABCG2* (rs2231142 G > **T**)	GG GT **TT**	82.3 (414) 16.5 (83) 1.2 (6)	28.4 (51) 51.4 (92) 20.1 (36)	56.5 (52) 31.5 (29) 12.0 (11)	56.7 (106) 35.3 (66) 8.0 (15)	75.5 (110) 23.1 (33) 1.4 (2)	68.0 (83) 28.7 (35) 3.3 (4)	48.9 (89) 40.1 (73) 11 (20)
*SLC2A9* (rs734553 G > **T**)	GG GT **TT**	5.6 (28) 37.8 (190) 56.6 (285)	– 2.3 (4) 97.7 (172)	– 3.2 (3) 96.8 (90)	– 1.1 (2) 98.9 (183)	1.4 (2) 15.4 (22) 83.2 (119)	– – 100 (121)	– 3.3 (6) 96.7 (176)
*SLC17A1* (rs1183201 **T** > A)	AA AT **TT**	23.1 (116) 46.1 (232) 30.8 (155)	4.0 (7) 33.7 (59) 62.3 (109)	1.1 (1) 27.0 (24) 71.9 (64)	3.8 (7) 26.6 (49) 69.6 (128)	11.8 (17) 44.4 (64) 43.8 (63)	29.7 (35) 55.0 (65) 15.3 (18)	6.6 (12) 43.7 (79) 49.7 (90)
*SLC16A9* (rs2242206 G > **T**)	GG CT **TT**	54.9 (276) 37.0 (186) 8.1 (41)	32.9 (59) 44.1 (79) 23.0 (41)	15.2 (14) 51.1 (47) 33.7 (31)	16.9 (31) 55.2 (101) 27.9 (51)	34.7 (50) 40.3 (58) 25.0 (36)	31.9 (38) 47.1 (56) 21.0 (25)	33.0 (60) 55.5 (101) 11.5 (21)
*GCKR* (rs780094 C > **T**)	CC CT TT	33.6 (169) 50.7 (255) 15.7 (79)	31.8 (57) 46.4 (83) 21.8 (39)	35.9 (33) 44.6 (41) 19.5 (18)	18.8 (35) 46.3 (86) 34.9 (65)	41.7 (60) 48.6 (70) 9.7 (14)	41.3 (50) 46.3 (56) 12.4 (15)	48.1 (88) 42.6 (78) 9.3 (17)
*SLC22A11* (rs17300741G > **A**)	**AA** AG GG	23.5 (118) 45.5 (229) 31.0 (156)	73.6 (128) 23.5 (41) 2.9 (5)	80.2 (73) 18.7 (17) 1.1 (1)	71.7 (129) 26.1 (47) 2.2 (4)	54.0 (75) 36.7 (51) 9.3 (13)	53.3 (64) 32.5 (39) 14.2 (17)	62.6 (112) 31.8 (57) 5.6 (10)
*SLC22A12* (rs505802 **C** > T)	**CC** CT TT	9.9 (50) 38.8 (195) 51.3 (258)	61.2 (112) 34.4 (63) 4.4 (8)	63.4 (59) 32.3 (30) 4.3 (4)	67.7 (126) 28.0 (52) 4.3 (8)	35.8 (52) 53.1 (77) 11.1 (16)	90.9 (110) 8.3 (10) 0.8 (1)	48.9 (90) 39.1 (72) 12.0 (22)
*LRRC16A* (rs742132 G > **A)**	**AA** AG GG	48.3 (243) 41.4 (208) 10.3 (52)	48.9 (88) 41.7 (75) 9.4 (17)	60.4 (55) 35.2 (32) 4.4 (4)	62.4 (116) 31.7 (59) 5.9 (11)	30.2 (44) 56.8 (83) 13.0 (19)	51.7 (62) 36.7 (44) 11.6 (14)	25.2 (46) 52.8 (96) 22.0 (40)
PDZK1** (rs12129861 **C** > T)	**CC** CT TT	30.4 (153) 47.3 (238) 22.3 (112)	42 (71) 5.4 (9) 52.6 (89)	54.3 (44) 4.9 (4) 40.8 (33)	47.8 (87) 2.2 (4) 50.0 (91)	36.3 (49) 5.9 (8) 57.8 (78)	37.7 (43) 3.5 (4) 58.8 (67)	42.2 (72) 8.7 (15) 49.1 (84)

The bolded letter refers to the risk allele linked to hyperuricemia or gout

^†^Data from the 1000 Genomes Project Phase III

^**^Allele deviated from HWE in all study subgroups

The prevalence of the A allele of rs1183201 (**T** > A) in *SLC17A1* was lower in Asian subgroups, Native Hawaiian, and Samoan than EUR descent. Marshallese, however, had a significantly higher prevalence (57.2%) than EUR (46.1%) (*p* = 0.002). Amongst the Asian subgroups, the frequency of the A allele of the rs1183201 (**T** > A) in *SLC17A1* was 2–3-folds lower than that in EUR (14.6%, 17.1%, and 20.9% for Koreans, Japanese, and Filipinos respectively vs. 46.1%). The A allele of the rs17300741G > **A** in *SLC22A11* in Asian subgroups of Korean, Filipino, and Japanese, was about twofold higher than EUR (89.6%, 85.3%, and 84.7%, respectively vs. 46.2% in EUR (*p* < 0.00001).

The frequency of the A allele of rs17300741G > **A** in *SLC22A11* was higher in Samoan, Native Hawaiian, and Marshallese than EUR (78.5%, 72.3%, and 70%, respectively, vs. 46.2% in EUR (*p* < 0.00001). In *SLC16A9*, the Asian subgroups (Koreans, Japanese, and Filipinos) had the highest prevalence of the T allele, which is approximately two times higher than EUR (59.2%, 55.5%, and 45%, respectively, vs.26.6%, *p* < 0.00001). Additionally, the prevalence of the risk allele T in Native Hawaiian (45.1%), Marshallese (44.5%), and Samoan (39.3%) were significantly higher compared with EUR (26.6%) (*p* < 0.00001). The remaining allele frequencies across all subgroups are summarized in Tables 3 and 4.

In the Japanese cohort, eight out of the eight uric acid alleles significantly differed from EUR (Table 5). All these eight alleles (100%) were prevalent in the Japanese population from EUR and were considered risk alleles. These risk alleles included: rs1183201 **T** > A in *SLC17A1*, rs2231142 G > **T** in *ABCG2*, rs2242206 G > **T** in *SLC16A9*, rs505802 **C** > T in *SLC22A12***,** *rs*734553 G > **T** in *SLC2A9*, rs17300741G > **A** in *SLC22A11,* rs742132 **A** > G in *LRRC16A*, and rs780094 C > **T** in *GCKR*.

**Table 5:** Summary of Total Risk Alleles across Asian, Native Hawaiian, and Pacific Islanders Subgroups

	EUR	Japanese	Korean	Filipino	Marshallese	Native Hawaiian	Samoan
Total alleles significantly different from EUR	Reference (8 SNPs)	100% (8/8)	75% (6/8)	75% (6/8)	75% (6/8)	75% (6/8)	100% (8/8)
HU or/gout risk allele index*		8	5	6	4	4	5
Percentage of risk allele*		100% (8/8)	83.5% (5/6)	100% (6/6)	66.5% (4/6)	66.5% (4/6)	62.5% (5/8)

^*^Indicates the risk allele that contributes to hyperuricemia or gout

*EUR* European

In the Korean cohort, six out of the eight uric acid SNPs significantly differed from EUR (Table 5). Five out of the six alleles (83.5%) were more prevalent in Korean than EUR and were considered risk alleles. These risk alleles genes/SNPs included: rs1183201 **T** > A in *SLC17A1*, rs2242206 G > **T** in *SLC16A9*, rs505802 **C** > T in *SLC22A12***, **rs734553 G > **T** in *SLC2A9*, rs17300741 G > **A** in *SLC22A11***.**

In the Filipino cohort, six out of the eight uric acid SNPs significantly differed from EUR (Table 5). All these six SNPs in Filipino were more prevalent (100%) than EUR and were considered risk alleles. These genes/SNPs included: rs1183201 **T** > A in *SLC17A1*, *rs*2231142 G > **T** in *ABCG2*, rs2242206 G > **T** in *SLC16A9*, rs505802 **C** > T in *SLC22A12***, **rs734553 G > **T** in *SLC2A9*, and rs17300741 G** > A** in *SLC22A11*.

In the Marshallese cohort, six out of the eight uric acid SNPs significantly differed from EUR (Table 5). Among those six SNPs, the Marshallese population had four uric acid alleles significantly more prevalent (66.5%) than EUR and were considered risk alleles. These genes/SNPs included: rs2231142 G > **T** in *ABCG2*, rs2242206 G > **T** in *SLC16A9*, rs505802 **C** > T in *SLC22A12,* and rs734553 G > **T** in *SLC2A9.*

In the Samoan cohort, eight out of the eight urate SNPs significantly differed from EUR (Table 5). Among those eight SNPs, five uric acid alleles (62.5%) had a higher prevalence in the Samoan population than EUR and were considered risk alleles. These genes/SNPs included: rs2231142 G > **T** in *ABCG2*, rs505802 **C** > T in *SLC22A12***, **rs734553 G > **T** in *SLC2A9*, rs17300741 G > **A** in *SLC22A11,* rs1183201 **T** > A in *SLC17A1.*

In the Native Hawaiian cohort, six out of the eight uric acid SNPs were significantly different from EUR (Table 5). Four out of six alleles (66.5%) were more prevalent in the Native Hawaiian population than EUR, and were considered risk alleles. These genes/SNPs include: rs505802 **C** > T in *SLC22A12***,** rs734553 G > **T** in *SLC2A9*, rs17300741 G > **A** in *SLC22A11,* and rs1183201 **T** > A in *SLC17A*. Among all our studied cohorts, Japanese, Filipino, and Korean subgroups had the highest HU and gout risk allele indices of 8, 6, and 5, respectively. The percentages of risk alleles were 100% in Japanese and Filipino, 83.5% in Korean, 66.5% in Native Hawaiian and Marshallese, and 62.5% in Samoan (Table 5).

### Genetic analysis and quality control

As a measure of quality control, genetic results were assessed for Hardy–Weinberg Equilibrium (HWE), using chi-square with Bonferroni corrected *p* < 0.006 for significance (Table 6). SNPs call rates were evaluated and reported for each ethnic group and the overall study cohort (n = 1059 participants). Overall SNPs call rate were 97.4% in *SLC22A12*, 95.1% in *SLC17A1,* 96% in *SLC16A9*, 96.9% in *ABCG2, *94.3 in *SLC22A11*, 91% in *PDZK1*, 96.1% in *SLC2A9,* 96.4*%* in *LRRC16A,* and 96.7% in *GCKR (*Table 7).

**Table 6:** Hardy Weinberg Equilibrium (HWE) Assessment of Targeted SNPs

Gene/SNP	Filipino	Japanese	Samoan	Marshallese	Native Hawaiian	Korean
*SLC17A1* (rs1183201)	0.7789	0.4036	0.3326	0.1743	0.9035	0.4449
PDZK1** (rs12129861)	0.0000	0.0000	0.0000	0.0000	0.0000	0.0000
*SLC22A11* (rs17300741)	0.4439	0.9076	0.4465	0.0109	0.3224	0.9926
*ABCG2* (rs2231142)	0.6376	0.3044	0.3942	0.8952	0.7880	0.0407
*SLC16A9* (rs2242206)	0.1473	0.1129	0.0275	0.6046	0.0250	0.5789
*SLC22A12* (rs505802)	0.8183	0.3809	0.2042	0.1753	0.1123	0.9398
*SLC2A9* (rs734553)	0.8788	0.9410	0.8211	0.0000	0.4077	0.8743
*LRRC16A* (rs742132)	0.8602	0.3476	0.4492	0.1642	0.0384	0.8089
*GCKR* (rs780094)	0.3981	0.4903	0.9620	0.9112	0.3209	0.4184

^**^ Indicates  deviation from HWE *p* < 0.006

**Table 7:** SNPs Call Rate (%)

	*SLC22A12* (rs505802)	*SLC17A1* (rs1183201)	*SLC16A9* (rs2242206)	*ABCG2 (rs*2231142)	*SLC22A11* (rs17300741)	*PDZK1* (rs12129861)	*SLC2A9* (rs734553)	*LRRC16A* (rs742132)	*GCKR* (rs780094)
Filipino	96.3	92.1	94.2	94.2	91.5	88.9	92.6	94.7	94.7
Japanese	98.4	97.3	96.8	98.9	95.2	96.2	97.8	98.4	98.4
Korean	97.9	93.6	95.9	96.8	95.7	85.2	97.8	95.7	96.8
Native Hawaiian	99.3	98.6	98..6	99.3	95.2	92.4	97.9	98.6	98.6
Marshallese	93.8	91.4	92.2	94.6	93.0	88.3	93.7	93.0	93.7
Samoan	98.4	96.7	97.3	97.3	97.3	91.4	97.3	97.3	97.8
Overall	97.4	95.1	96.0	96.9	94.3	91.0	96.1	96.4	96.7

## Discussion

Our study found that the post-partum women of Asian ancestry had a higher prevalence of HU or gout risk alleles when compared to EUR. Specifically, all significantly different uric acid alleles between the Japanese and Filipino cohorts compared with EUR, were all considered HU or gout risk alleles. These results could partially explain the differential prevalence of HU and gout across different ethnic and racial groups based on their genetic makeup. Therefore, a discussion on the role of these various genes/alleles in developing HU or gout is warranted.

*ABCG2* gene encodes the ATP-Binding Cassette G-protein transporter located on the apical membrane in the proximal renal tubule, and it is expressed in the gastrointestinal tract and liver. ABCG2 is a major urate excretion transporter [19]. Genetic polymorphisms in the *ABCG2* were reported to contribute to elevated urate levels leading to HU and gout. The risk allele T of the rs2231142 G > **T** (Q131K) in *ABCG2* is associated with increased urate levels. Individuals with the TT genotype are at higher risk for HU and gout than their GG counterparts. A recent study reported that the T allele is 3- times higher in East Asians than in EUR. This prevalence suggests that East Asian populations are at higher risk for developing HU and gout [20].

The genetic polymorphism rs22131142 (G > **T**) in *ABCG2* is significantly associated with a higher risk for HU and gout among different populations [21–23]. A study conducted in a Korean cohort showed that the rs22131142 (G > **T)** is strongly associated with gout risk (Odds ratio [OR] 3.32; 95% confidence interval [CI]: 2.11 to 5.20) [24]. Also, a study of 6881 Koreans identified that the genetic polymorphism rs2231142 (G > **T**) was associated with increased SU levels (Effect size = 0.220, *p* = 2.06E-29) [25]. Consistent with our results, a study reported that the minor allele frequency (MAF) of the risk allele T of the rs2231142 in *ABCG2* was high in Japanese and Koreans compared to Caucasians (0.29, 0.28, vs. 0.11) [26]. Additionally, a meta-analysis conducted on a multi-ethnic cohort reported that the T allele of the rs22131142 (G > **T)** in *ABCG2* was strongly associated with HU and gout across different ethnic groups and further modulates the severity and gout onset [27]. While genetic polymorphism in *ABCG2* increases the risk for developing gout in the population at large, genetic polymorphism in *ABCG2* exerts different effect sizes between sexes. Specially, gene-sex interaction exists for *ABCG2*, with effect allele exerting greater influence on gout risk in men than women [28].

Overall, the genetic polymorphism rs2231142 (G > **T**) in the *ABCG2* gene is considered the most significant genetic polymorphism in developing HU or gout in multiple populations compared with other uric acid risk alleles. Sun et al. evaluated the impact of 11 genetic loci of which *ABCG2* rs2231142 (G > **T**) was one of the genes associated with serum urate concentrations in the Chinese population [29]. Also, Zhang et al. reported that the SNP rs2231142 of the *ABCG2 *gene was associated with HU in the American population consisting of EUR Americans, African Americans, Mexican Americans, and Indian Americans [30]. Our finding suggests that the rs2231142 (G > **T**) in *ABCG2* may increase urate levels and gout risk in Asian and Pacific Islander subgroups compared to EUR.

*SLC2A9* encodes the GLUT9 transporter with high-capacity for urate, fructose, and glucose (Table 2). *SLC2A9* is known to be strongly associated with urate regulation in the human body [31]. It is mainly expressed in the kidneys and liver, but it is also expressed in human articular cartilage [32]. The intronic polymorphism rs734553 (G > **T**) in *SLC2A9* is associated with an increased risk for HU and gout resulting from a change in transporter affinity for urate [33]. This genetic variation strongly affects SU levels in EUR ancestry, specifically EUR women (Effect size = 0.315, *p* = 5.22 × 10–201) [34]. Reginato Am et al*.* have identified that polymorphism rs734553 in the * SLC2A9* gene is linked to SU levels and gout in the Polynesian population [35]. The prevalence of the T allele of the rs734553 (G > **T)** in our Asian and NHPI subgroups was significantly higher than in EUR, suggesting that carrying the T allele will likely increase the risk of HU in both the Asian and NHPI subgroups.

*SLC17A1* encodes the voltage-gated human sodium-dependent phosphate co-transporter type 1 protein (NPT1), located on the proximal tubule's apical side in the kidney and works as renal urate efflux transporter. Decreased SU levels were found to be associated with the genetic polymorphism rs1183201 (**T** > A) in *SLC17A1* (Effect size =  − 0.062, 95% CI: − 0.078; − 0.459) with the effect allele A, as the protective allele in EUR descent (Table 2). The polymorphism rs1183201 (**T** > A) in *SLC17A1*was associated with decreased SU levels with a prevalence of 48.2% in EUR descent [34]. Amongst our Asian subgroups, the frequency of the A allele in the rs1183201 (**T** > A) was 2–3-folds lower than EUR (Table 3). The significant differences in the A allele frequencies across the population subgroups, mostly Asian subgroups, suggest that some ethnic groups could be inherently predisposed to high urate levels.

*SLC22A12* encodes URAT1 transporter, a protein expressed on the kidney's apical side of the proximal convoluted tubule. This transporter is responsible for the majority of the urate reabsorption and is a primary target for urate-lowering therapies [36]. A previous study reported that the loss of function of URAT1 could cause hypouricemia in a Japanese cohort, suggesting that URAT1 plays an essential role in regulating the urate tubular reabsorption [37]. The intergenic polymorphism rs505802 (**C** > T) in *SLC22A12* was observed to reduce urate levels in individuals of EUR ancestry. Specifically, the T allele was associated with lower SU levels in women and men (Effect size =  − 0.073 and − 0.047, respectively) [34]. Jang et al*.* reported that the T6092C genetic variant in *SLC22A12* was also significantly associated with SU concentration amongst the male Korean population [38]. The T6092C at rs1529909 of *SLC22A12* was found in nearly complete linkage disequilibrium with rs505802 of *SLC22A12* (D’ = 0.99, R^2^ = 0.98). The prevalence of the T allele in our population subgroups was significantly lower than EUR, with 3–4-folds lower in Asian and NHPI subgroups (Table 3). These findings suggest that Asian and NHPI population subgroups could have higher baseline urate levels compared with EUR. The frequency of the C allele in Marshallese, however, was more than three times compared to EUR (95% vs. 29.3%, *p* < 0.00001). These results further suggest a higher risk for HU and gout in Marshallese and a possible implication in the response to treatments targeting URAT1 transporter in Asian and NHPI subgroups.

*SLC22A11* is predominantly expressed on the proximal tubule's apical side in the kidney and encodes the organic anion transporter 4 (OAT4) (Table 2). The primary role of the organic anion transporter 4 (OAT4) is urate reabsorption. Like the URAT1 transporter, OAT4 is a target for urate-lowering therapy [39]. The SNP rs17300741 (G > **A**) in the *SLC22A11* was associated with renal urate underexcretion type gout in the Japanese population (*p* = 0.049) [40]. Kolz et al. have also reported a significant association between the polymorphism rs17300741 G > **A** in *SLC22A11* and urate levels in individuals of Caucasian descent (*p* = 6.7 × 10 − 14) [34]. Moreover, previous reports demonstrated the association between the rs17300741 G > **A** in gout prevalence, which is two-fold higher in non-EURs relative to EUR [41]. Our analysis found that the prevalence of the A allele was higher across all selected subgroups than EUR (Table 3). Our analysis of the rs17300741 G > **A** in *SLC22A11* suggests a higher genetic risk for HU or gout in Asian and NHPI subgroups compared with EUR. Collectively, our analysis demonstrates that the frequencies of risk alleles C and A in both loci-*SLC22A12* and *SLC22A11,* respectively*,* were significantly higher in Filipino, Korean, Japanese, Samoan, Marshallese, and Native Hawaiian relative to EUR (Table 3). Notably, the prevalence of risk alleles rs505802 (**C** > T) of *SLC22A12* and rs17300741 (G** > A**) of *SLC22A11* genes were highest in Asian subgroups compared with the NHPI population.

*SLC16A9* encodes for monocarboxylic acid transporter protein across the cell membrane (MCT9). It is located on the proximal tubule's apical side of the kidney and is responsible for urate excretion. A missense variant rs2242206 (G > **T**) in the *SLC16A9* has been reported to dysregulate the urate level (Table 2). The exact function of the MCT9 transporter is still unclear. Nonetheless, Nakayama et al. have found a significant relationship between the rs2242206 G > T (K258T) in *SLC16A9*, and gout (*p* = 0.012), with 1.28 OR in a Japanese population [42]. Our cohort analysis showed that the frequency of T allele across the subgroups was significantly higher than that of EUR ancestry (Table 3). These findings suggest that individuals of Asian descent carrying the polymorphism rs2242206 (G > **T**) in *SLC16A9* could be at higher risk for and more susceptible to gout, especially in individuals of Japanese, Korean, and Filipino descent.

*GCKR* encodes glucokinase regulatory protein (GCKR), which exerts a significant role in developing the components of the metabolic syndrome, including triglyceride levels and glucose metabolism (Table 2) [43, 44]. Earlier studies have demonstrated the relationship between urate levels and metabolic syndrome-related traits such as insulin resistance and hypertension through oxidative stress and inflammatory pathway [34]. The SNP rs780094 (C > **T**) in *GCKR* is strongly associated with gout in the male Han-Chinese population [45]. Furthermore, the T allele of the rs780094 C > **T** is associated with urate concentration in EUR ancestry [34]. Meanwhile, the MAF of the C allele was higher in the Korean cohort compared with Caucasians (0.47 vs. 0.42) [26]. In our analysis, the frequency of the T allele was higher only in the Japanese cohort than EUR (58% vs. 41.1%, *p* < 0.00001) and only lower in Samoan than EUR (30.6 vs. 41.1%, *p* < 0.006) (Table 3). There was no significant difference in the T allele between Filipino and Korean compared to EUR. These results suggest that Japanese individuals could be at a higher risk for developing HU, gout, and gout-related comorbidities compared with EUR. Noteworthy, the GCKR protein is associated with modulating some metabolic functions; hence, this finding might partially suggest a biological mechanism for developing cardiometabolic disorders, including HU and gout. Theoretically, the SNP rs780094 (C > T) could be a plausible candidate to study the intersection of urate levels and the excess risk of developing both gestational diabetes and hypertension among pregnant women of Asian ancestry.

*LRR16A* is expressed on the apical side of the proximal tubule in the kidney and encodes the capping protein ARP2/3 and myosin-I linker (CARMIL) (Table 2). This protein has a role in urate transportome formation, which mediates urate reabsorption [34, 46]. Hiraka Ogata et al. have found a significant association between the intergenic variant homozygote AA in rs742132 (G > **A**) in *LRRC16A* and the risk of gout disease among Japanese males [47]. Moreover, a genetic variant rs742132 (G > **A**) in *LRRC16A* is associated with increased SU in EUR ancestry [34]. Notably, a GWAS conducted on an East Asian population, including Korean, showed that rs742132 in *LRRC16A* is associated with elevated urate levels [48]. Amongst our population subgroups, only the Japanese cohort had a significant difference and the highest frequency of the A allele compared to EUR (78.2%, vs. 69%, *p* = 0.0009). On the other hand, the Samoan cohort had a significantly lower frequency of the A allele compared with EUR (51.7%, vs. 69%, *p* < 0.00001) (Table 3). Asian subgroups of Japanese and Korean had the highest A allele frequency compared to all other subgroups in this study, which are consistent with previous reports [49]. Our findings suggest that the genetic polymorphism rs742132 (G > **A**) in *LRRC16A* may explain the differential prevalence of HU or gout risk in Asian subgroups.

Our results have collectively shown that the frequency of HU or gout risk alleles in several population subgroups significantly differs from EUR (*p* < 0.006). We found that the Asian subgroups had the highest HU or gout risk allele prevalence compared with the NHPI subgroups. These results are consistent with the patient claims data of the ambulatory care clinics that gout diagnosis in the Asian population living in the U.S. is about three times more than EUR. Consistent with the previous reports, our results provide more evidence that individuals of Asian descent have a higher risk for developing HU or gout than EUR [22, 50, 51]. We believe our analysis has broadened our understanding of gout genetic risk in select underrepresented population subgroups in biomedical research, allowing for greater delineation of populaiton-specific risk for HU or gout.  

### Limitations

First, the study was a retrospective analysis conducted only on pregnant women. Therefore, prospective representative samples of the population are needed to validate our findings. Second, we believe that other factors such as certain social and dietary habits, older age, and male sex significantly contribute to developing HU and gout; therefore, our participants represented the lowest risk profile for gout development, explaining the lack of gout diagnosis in our study cohort. Nonetheless, our genetic assessment of HU and gout are directionally consistent with the epidemiology of gout risk in certain racial groups. Third, we had a limited number of SNPs in our analysis, recognizing that other genes/SNPs are associated with the development of HU and gout. However, our limited SNPs were based on robust GWAS. Fourth, our study participants did not have measured SU levels to conduct an association analysis between genotype and phenotype. Finally, some of our subgroups could have benefited from larger sample sizes to estimate specific risk allele frequencies. Therefore, we believe future studies and broader genetic assessment are needed.

## Conclusions

Our analysis identified that HU and gout risk alleles were significantly more frequent in Asian subgroups (Japanese, Filipino, and Korean) than in EUR. These findings are consistent with our previous report suggesting that Japanese and Han-Chinese populations had the highest prevalence of gout or HU risk alleles than EUR [22]. Our genetic assessment of HU and gout risk alleles in Asian, Native Hawaiian, and Pacific Islander subgroups highlights the distinct within-population differences, especially among subgroups generally aggregated as one race or ethnicity. Consistent with gout epidemiology, pregnant women and childbearing age women are unlikely to develop gout, despite having the genetic risk. To this end, this study reported no gout cases among our study participants.

### Clinical research perspective

Genetic assessment in ancestral subgroups could play a crucial role in addressing ongoing health disparities, including gout and gout-related comorbidities. Our results not only suggest that genetic data may explain gout epidemiology but may be utilized to improve patient treatment outcomes by predicting disease risk and selecting the proper treatment. Indeed, this study is the first genetic investigation focusing on several urate genes/SNPs pairs in multiple underrepresented ancestral subgroups of pregnant women. Our findings could advance future research to assess the effects of hormonal changes during pregnancy on urate genes expression and the role of HU and gout risk alleles in pregnancy outcomes such as gestation diabetes, gestation hypertension, and pre-eclampsia.

## Data Availability

All applicable and relevant data are included in the paper. Access to the raw data and biospecimens can be directed to the University of Hawaii Biorepository (http://uhbio.jabsom.hawaii.edu/Access.html).

## Abbreviations

SU: Serum urate
HU: Hyperuricemia
SNP: Single nucleotide polymorphism
NHANES: National Health and Nutrition Examination Survey
GWAS: Genome-Wide Association Studies
EUR: European
NHPI: Native Hawaiian and Pacific Islander
CVD: Cardiovascular disease
IR: Insulin resistance
MAF: Minor allele frequency
HWE: Hardy–Weinberg equilibrium
